# Comparing Single-Incision Cholecystectomy and Multiple-Incision Cholecystectomy: A Systematic Review and Meta-Analysis of Postoperative Complications

**DOI:** 10.7759/cureus.78434

**Published:** 2025-02-03

**Authors:** Diya Linkwinstar, Anu Shine, Haiqa Naseem, Syeda Zara, Siti Jabbir, Kapilraj Ravendran

**Affiliations:** 1 Internal Medicine, Medical University Sofia, Sofia, BGR; 2 General Surgery, Gradscape, London, GBR; 3 Internal Medicine, Medical University Pleven, Pleven, BGR; 4 Trauma and Orthopaedics, Bedfordshire Hospital NHS Foundation Trust, Luton, GBR; 5 General Surgery, The Hillingdon Hospital NHS Foundation Trust, Uxbridge, GBR; 6 Trauma and Orthopaedics, East and North Hertfordshire NHS Trust, London, GBR

**Keywords:** meta-analysis, multiport laparoscopic cholecystectomy, postoperative pain, recovery, single-incision laparoscopic cholecystectomy, wound infection

## Abstract

Laparoscopic cholecystectomy is the gold standard for managing benign gallbladder disease, with the conventional multiport technique widely practiced. Single-incision laparoscopic cholecystectomy (SILC) has emerged as an alternative, offering potential benefits such as improved cosmetic outcomes, reduced pain, and quicker recovery; however, its efficacy and safety compared to conventional laparoscopic cholecystectomy (CLC) remain unclear. This meta-analysis, which followed Preferred Reporting Items for Systematic Reviews and Meta-Analyses (PRISMA) guidelines, included 51 studies with 2,069 patients to compare clinical outcomes such as postoperative complications, pain, recovery time, and wound infection rates between SILC and CLC. SILC was associated with slightly higher postoperative pain scores (mean difference, 0.18; 95% CI, 0.09-0.27; p < 0.001), increased wound infection rates (OR, 1.77; 95% CI, 1.30-2.79; p < 0.001), and a marginally longer hospital stay (mean difference, 0.22 days; 95% CI, 0.16-0.28; p < 0.001). Recovery time showed no significant difference (mean difference, 0.01 days; 95% CI, -0.56 to 0.59; p = 0.73). While SILC offers a cosmetic advantage due to fewer incisions, it is associated with marginally less favorable clinical outcomes compared to CLC, highlighting the need for further research to assess its long-term efficacy and refined surgical techniques.

## Introduction and background

Laparoscopic cholecystectomy is the gold standard for treating benign gallbladder issues. Around 70% of emergency cholecystectomies and 90% of elective cholecystectomies can be performed with this method [1]. Laparoscopic cholecystectomy is a combined endoscopic-operative method for removing the gallbladder. This surgery is available to patients who have gallstones that are causing symptoms. It is carried out through four cannulas and is directed by an endoscope, camera, and video monitor [2]. Under a monitor, the gallbladder is removed from the hepatic bed. Potential side effects include bleeding, damage to the common bile duct, and technical issues, including gallbladder perforation [2].

Three or four ports (typically four) are employed in the multiport technique used in traditional laparoscopic cholecystectomy [3]. Traditionally, a camera and a clip applier may be accessed through two ports that are 10 mm in size [3]. Two 5-mm ports are used to manipulate the gallbladder to adequately expose the surgical field [4].

A subsequent development that improved the laparoscopic technique using a single port for access was single-incision laparoscopic cholecystectomy (SILC) [5]. A single incision, usually made in the patient's navel, is used to execute the whole process in single-incision laparoscopic surgery, often referred to as single-port surgery or SILS [6]. In contrast to conventional laparoscopic procedures, which need several incisions, SILS seeks to reduce scarring and enhance cosmetic results while preserving the surgical intervention's effectiveness [6].

As of now, the benefits of SILS remain unclear. According to some theories, SILS may offer less postoperative discomfort, a quicker return to work, fewer port-site problems, and better cosmesis [7].

Our study compares single-incision to conventional multiport incision laparoscopic cholecystectomy techniques through a meta-analytic systematic review, assessing their relative efficacy and safety.

## Review

Methodology

Search Strategy

The objective of this study was to compare the outcomes of SILC with conventional laparoscopic cholecystectomy (CLC) in surgical procedures and postoperative complications. The study protocol was collaboratively developed and agreed upon by all authors, following the PRISMA (Preferred Reporting Items for Systematic Reviews and Meta-Analyses) guidelines to ensure a systematic approach [8].

Five researchers (D.L., A.S., H.N., Z.S., and S.J.) reviewed all extracts. A comprehensive literature search was conducted using the following databases: Google Scholar, PubMed, Cochrane Library, Scopus, Embase, and MEDLINE. No restrictions were applied during the search; however, the studies included in the review were published between 2010 and 2023. The search aimed to identify relevant studies using the following keywords: "single-incision laparoscopic cholecystectomy" OR "SILC" OR "single-port cholecystectomy" AND "conventional laparoscopic cholecystectomy" OR "CLC" OR "multi-port laparoscopic cholecystectomy" AND "postoperative complications" OR "surgical complications" OR "complications." The senior author (K.R.) reviewed the results.

Study Selection and Eligibility Criteria

The selection process followed predefined inclusion and exclusion criteria. Two researchers (D.L. and A.S.) reviewed the full-text studies. A third researcher (S.J.), who reviewed impartial studies, was responsible for screening studies to ensure adherence to inclusion criteria and minimize potential bias in study selection.

Quality assessment of included studies was conducted using the ROB-2 tool [9] to assess the risk of bias in randomized trials and the ROBINS-I tool [10] to assess the risk of bias in non-randomized studies. Results were then visualized using the risk of bias visualization (ROBVIS) tool [11] for clarity and consistency in presentation.

Eligible study types included prospective, retrospective, randomized, and non-randomized controlled trials. The intervention criteria focused on SILC versus CLC. The outcomes assessed included postoperative and surgical complications.

Studies were excluded if they comprised conference abstracts, were published in languages other than English, lacked full-text availability online, or were editorials and review articles.

Data Extraction and Analysis

Data extraction was independently performed by two reviewers (H.N. and Z.S.) to ensure accuracy and consistency. Discrepancies were resolved through discussion or consultation with a third reviewer (S.J.). The extracted data included study characteristics such as authorship, publication year, journal, sample size, and study design. Information on the type of intervention (SILC or CLC) was also extracted.

Reported outcomes included postoperative complications and recovery metrics, such as hospital stay (mean number of days and standard deviation), postoperative pain scores, conversion rates, recovery time to normal activities (mean number of days and standard deviation), and wound site infection rates. Additional baseline characteristics, including the number of patients, surgical techniques, gender distribution, body mass index (BMI), and procedural indications, were also recorded.

The results were reviewed and validated by a senior researcher (K.R.). The review protocol was registered with the PROSPERO database under the identification number CRD42024595719.

Statistical Analysis

This meta-analysis was conducted by D.L. and A.S. and was reviewed by the senior researcher (K.R.) in accordance with the PRISMA statement and the criteria of the Cochrane Collaboration. Binary endpoints were compared using odds ratios (OR) with 95% confidence intervals, while continuous outcomes were pooled using weighted mean differences. Heterogeneity was evaluated using the Cochran Q test and I² statistics, with p-values < 0.10 and I² < 25% considered significant. The statistical analysis was conducted using Review Manager 5.4 (Nordic Cochrane Centre, The Cochrane Collaboration, Copenhagen, Denmark).

Results

**Figure 1:**
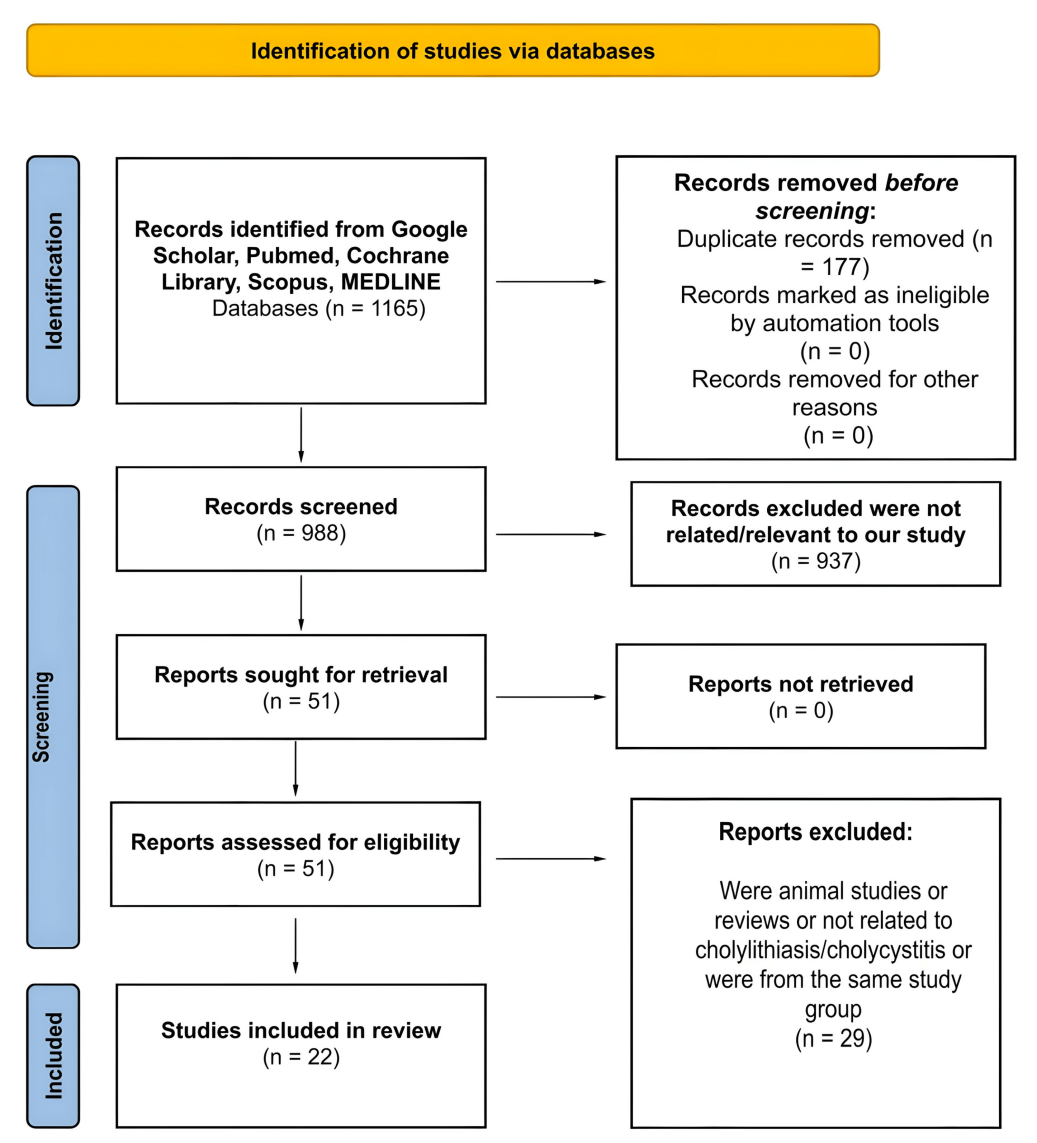
PRISMA flow diagram demonstrating the literature selection strategy. PRISMA: Preferred Reporting Items for Systematic Reviews and Meta-Analyses.

The initial database search identified 1,165 articles, of which 177 duplicates were removed. After applying the exclusion criteria, 937 studies were excluded, leaving 51 articles that initially met the inclusion criteria. These articles were then thoroughly reviewed for relevance to the study’s objectives, resulting in the exclusion of 29 studies due to insufficient detail or lack of specific relevance. Ultimately, 22 studies were included in the final review. Figure 1 displays a PRISMA-style graphic illustrating the study selection process [8].

Table 1 summarizes the overall findings from the reviewed studies, which included 22 publications analyzing the outcomes of SILC compared to CLC [12-33].

**Table 1:** Overall findings from the reviewed studies, which included 22 publications analyzing the outcomes of single-incision laparoscopic cholecystectomy (SILC) compared to conventional laparoscopic cholecystectomy (CLC). SILC was found to have comparable clinical outcomes to CLC in terms of hospital stay and postoperative pain scores but showed a slightly higher rate of wound site infections in several studies [13,17,26]. For instance, certain trials reported higher infection rates with SILC (e.g., 12% vs. 6%), although it offered the benefit of reduced postoperative pain in select cases [13,19]. Both techniques were effective in achieving satisfactory recovery, with SILC providing notable cosmetic advantages due to the use of fewer incisions [16,18,25]. However, CLC consistently demonstrated a lower overall complication rate across studies [16,23,29]. These findings highlight SILC as a viable alternative for patients prioritizing minimal scarring, though further refinement of surgical techniques is needed to reduce its associated risks [14,22,28].

Author	Journal	Type of Study	Year of Study	Year of Publication	Country of Study	Number of Patients	BMI (kg/m^2^)	Hospital Stay (Mean No. of Days and SD)	Post-Op Pain Score	Conversion Rate	Wound Site Infection	Recovery Time (Mean No. of Days and SD)
Yilmaz et al., 2013 [12]	Ann Surg Treat Res	Prospective randomized study	2007-2009	2013	South Korea	SILC: 43	SILC: 24.2 ± 4.0	-	SILC: 3.23 ± 0.75	SILC: 2.3% (n=1)	SILC: 2.3% (n=1)	-
						CLC: 40	CLC: 23.3 ± 3.0		CLC: 2.95 ± 0.90	CLC: 2.5% (n=1)	CLC: 2.5% (n=1)	
Cao et al., 2011 [13]	Surg Laparosc Endosc Percutan Tech	Randomized clinical trial	2010	2011	China	SILC: 57	SILC: 28.6± 4.4	SILC: 2.1 ± 1.1	SILC: 2.3 ± 0.9	SILC: 3.5% (n=2)	SILC: 1.8% (n=1)	-
						CLC: 51	CLC: 29.1± 5.1	CLC: 2.8 ± 0.8	CLC: 2.6 ± 1.2	CLC: 0% (n=0)	CLC: 1.8% (n=1)	
Yong Chang et al., 2014 [14]	World J Surg	Randomized controlled trial	2010-2012	2014	Singapore	SILC: 50	SILC: 25.34 (4.50)	-	SILC: 2.254 (2.248)	SILC: 0% (n=0)	SILC: 2% (n=1)	SILC: 4 (1–14)
						CLC: 50	CLC: 25.83 (6.45)		CLC: 2.350 (2.513)	CLC: 0% (n=0)	CLC: 2% (n=1)	CLC: 3 (0–14)
Gupta et al., 2023 [15]	Int Surg J	Prospective randomized study	2017-2018	2023	India	SILC: 75	SILC: 22.566±2.78	-	SILC: 0.666±0.844	SILC: 0% (n=0)	-	-
						CLC: 75	CLC: 22.550±2.90		CLC: 0.900±0.884	CLC: 0% (n=0)		
Ma et al., 2011 [16]	Ann Surg	Randomized controlled trial	2009-2010	2011	USA	SILC: 21	SILC: 28.2 (5.3)	-	SILC: 5 (1.8)	-	SILC: 14% (n=3)	-
						CLC: 22	CLC: 30.7 (6.1)		CLC: 12 (1.8)		CLC: 18% (n=4)	
Hajong et al., 2016 [17]	J Clin Diagn Res	Prospective randomized comparative study	2014-2015	2016	India	SILC: 32	SILC: 65.6	SILC: 1.72 ± 0.40	SILC: 2	SILC: 0% (n=0)		-
						CLC: 32	CLC: 59.4	CLC: 2.468 ± 0.59	CLC: 3	CLC: 0% (n=0)		-
Deveci et al., 2013 [18]	J Korean Surg Soc	Prospective randomized study	2010-2012	2013	Turkey	SILC: 50	SILC: 27.90 ± 4.96 (18–40)	SILC: 1.06 ± 0.23 (1–2)	-	SILC: 8.0% (n=4)	SILC: 4.0% (n=2)	-
						CLC: 50	CLC: 28.10 ± 5.22 (19–42)	CLC: 1.04 ± 0.19 (1–2)		CLC: 12.0% (n=6)	CLC: 4.0% (n=2)	
Malladad and Kulkarni, 2018 [19]	Int Surg J	Comparative randomized study	2017-2018	2018	India	SILC: 30	-	SILC: 4.04±1.34	SILC: 4.42	SILC: 12% (n=4)	SILC: 4.04	SILC: 3.90
						CLC: 30		CLC: 3.93±1.14	CLC: 16.07	CLC: 10% (n=3)	CLC: 3.93	CLC: 0
Chow et al., 2010 [20]	Arch Surg	Prospective, retrospective, randomized	2007-2008	2010	England	SILC: 58	SILC: 26.6	SILC: 0.76	-	SILC: 0% (n=0)	SILC: 0% (n=0)	-
						CLC: 41	CLC: 28.2	CLC: 1.53		CLC: 0.25% (n=4)	CLC: 0.06 (n=1)	-
Seifeldin et al., 2022 [21]	Egypt J Surg	Prospective, randomized, controlled clinical trial	2019-2020	2022	Egypt	SILC: 20	SILC: BMI less than 35.	SILC: 2.38 ± 0.4	-	SILC: 10% (n=2)	-	-
						CLC: 20	CLC: BMI less than 35.	CLC: 2.47 ± 0.47		CLC: 0% (n=0)		
Krishna et al., 2020 [22]	Indian J Surg	Single-blinded, randomized study	2012-2014	2020	India	SILC: 47	SILC: 22.36 ± 2.38	SILC: 27.06 ± 7 h	SILC: 0.41 ± 0.49	-	-	SILC: 6
						CLC: 47	CLC: 22.10 ± 2.14	CLC: 25.21± 5 h	CLC: 0.40 ± 0.49			CLC: 3
Suh et al., 2023 [23]	Ann Hepatobiliary Pancreat Surg	Retrospective analysis, randomized control study	2014-2015	2023	South Korea	SILC: 58	SILC: 24.8 (23.1–27.3)	SILC: (1–3)	SILC: 2.2 ± 1.8	SILC: 1.7% (n=1)	SILC: 0% (n=0)	-
						CLC: 117	CLC: 24.2 (21.9–26.8)	CLC: (1–2)	CLC: 2.3 ± 1.7	CLC: 0.9% (n=1)	CLC: 0% (n=0)	
McGregor et al., 2011 [24]	J Gastrointest Surg	Non-randomized study	2010	2011	UK	SILC: 11	SILC: 27 ± 1.44	SILC: 0.97 (0.35)	SILC: 0 (0)	SILC: 27.3% (n=3)	SILC: 0% (n=0)	-
						CLC: 12	CLC: 31.72 ± 1.07	CLC: 0.86 (0.11)	CLC: 1 (4)	CLC: 0% (n=0)	CLC: 8.3% (n=1)	-
Balachandran et al., 2017 [25]	World J Surg	Retrospective analysis	2011-2014	2017	Unknown	SILC: 415	SILC: 29.0 (±6.1)	SILC: 1.9 (±3.1)	SILC: 35 (8.4)	SILC: 3.2% (n=13)	SILC: 3.9% (n=16)	SILC: 16 (3.9)
						CLC: 263	CLC: 30.5 (±7.8)	CLC: 2.4 (±2.3)	CLC: 11 (4.2)	CLC: 4.9% (n=13)	CLC: 1.1% (n=3)v	CLC: 3 (1.1)
Garg et al., 2012 [26]	Surg Laparosc Endosc Percutan Tech	Prospective controlled trial	-	2012	India	SILC: 35	SILC: 25.7± 4.3	SILC: 1.08± 0.6	SILC: 1.71± 1.8	SILC: 0% (n=0)	SILC: 7.81± 3.8	-
						CLC: 29	CLC: 26.4± 3.4	CLC: 1.21± 0.5	CLC: 2.00± 1.1	CLC: 0% (n=0)	CLC: 8.35± 2.2	
Hoyuela et al., 2019 [27]	Hernia	Cohort study	2009-2011	2019	Belgium	SILC: 42	SILC: 26.7±3.5	SILC: 1.16±0.43	-	SILC: 0% (n=0)	-	-
						CLC: 140	CLC: 27.4±3.8	CLC: 1.29±0.6		CLC: 1% (n=2)		-
van der Linden et al., 2015 [28]	World J Gastrointest Surg	Non-randomized trial	2011-2012	2015	Netherlands	SILC: 136	SILC: 27 (17-40)	SILC: 1	-	SILC: 1	-	-
						CLC: 659	CLC: 28 (19-46)	CLC: 2		CLC: 0		
Wong et al., 2012 [29]	Surg Laparosc Endosc Percutan Tech	Prospective case-control study	2009-2010	2012	Hong Kong	SILC: 20	SILC: 22.9± 2.8	SILC: 1 (1-3)	SILC: 2.9 ± 1.6	SILC: 0% (n=0)	SILC: 0% (n=0)	-
						CLC: 20	CLC: 23.6± 2.3	CLC: 1 (1-2)	CLC: 4.8± 1.5	CLC: 0% (n=0)	CLC: 0% (n=0)	-
Karim et al., 2012 [30]	Int J Surg	Retrospective review	2009-2011	2012	-	SILC: 45	SILC: 23.6	SILC: 22h	SILC: 0.73	SILC: 0% (n=0)	-	-
						CLC: 62	CLC: 29.1	CLC: 31h	CLC: 0.71	CLC: 0% (n=0)		-
Hosseini et al., 2015 [31]	Adv Biomed Res	Cross-sectional study, non-randomized	2011-2013	2015	Iran	SILC: 52	SILC: 23.97 ± 4.78	SILC: 1.56 ± 0.95	SILC: 5.84 ± 2.53	SILC: 1.9% (n=1)	SILC: 16.77 ± 16.6	-
						CLC: 62	CLC: 26.22 ± 4.67	CLC: 1.61 ± 1.23	CLC: 6.01 ± 4.79	CLC: 0% (n=0)	CLC: 16.55 ± 17.44	
Lee et al., 2018 [32]	Ann Hepatobiliary Pancreat Surg	Retrospective cohort study, non-randomized	2010-2016	2018	South Korea	SILC: 119	SILC: 24.7 ± 3.5	SILC: 3.5±3.5	-	-	SILC: 1.2% (n=12)	SILC: 2.7 ± 2.1
						CLC: 355	CLC: 24.9 ± 3.8	CLC: 2.7±2.1			CLC: 1.5% (n=16)	CLC: 3.5 ± 3.5
Casaccia et al., 2019 [33]	JSLS	Non-randomized retrospective cohort study	2016-2017	2019	Italy	SILC: 40	SILC: 27.2 ± 5	SILC: 1.9 ± 0.9	SILC: 1.69 ± 1.8	SILC: 0% (n=0)	SILC: 12.5% (n=5)	SILC: 1.9 ± 0.9
						CLC: 40	CLC: 27.0 ± 5.3	CLC: 2.3 ± 1.2	CLC: 1.68 ± 1.8	CLC: 0% (n=0)	CLC: 2.5% (n=1)	CLC: 2.3 ± 1.2

Conversion Rate

**Figure 2:**
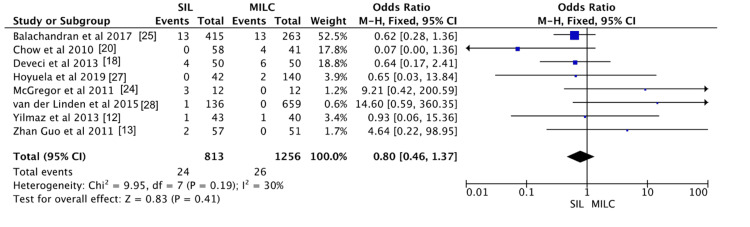
Meta-analysis comparing conversion rate between single incision laparoscopic cholecystectomy and conventional laparoscopic cholecystectomy.

Seven studies reported conversion rates for SILC and CLC. The SILC group had 24 conversions out of 813 patients (2.95%), while the CLC group had 26 conversions out of 1,256 patients (2.07%). The CLC group demonstrated a slightly lower conversion rate compared to SILC; however, the difference was not statistically significant (OR, 0.80; 95% CI, 0.46-1.37; p = 0.41; I² = 30%). The findings are shown in Figure 2.

Hospital Stay

**Figure 3:**
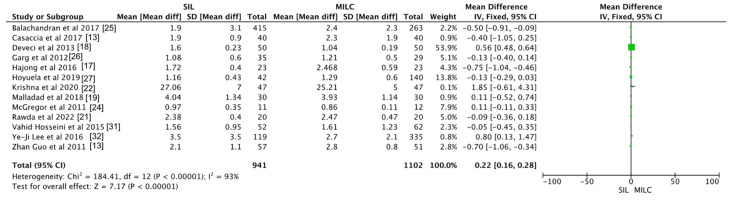
Meta-analysis comparing hospital stay between single incision laparoscopic cholecystectomy and conventional laparoscopic cholecystectomy.

Thirteen studies assessed the duration of hospital stay in the SILC and CLC groups. The mean hospital stay was slightly longer in the SILC group compared to the CLC group (mean difference, 0.22; 95% CI, 0.16-0.28; p < 0.00001). Heterogeneity across studies was high (I² = 93%). The results are illustrated in Figure 3.

Recovery time

**Figure 4:**
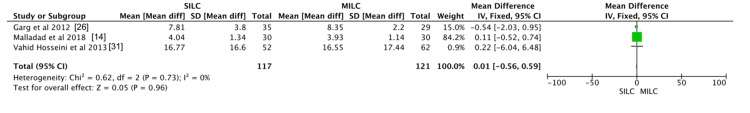
Meta-analysis comparing recovery time between single incision laparoscopic cholecystectomy and conventional laparoscopic cholecystectomy.

Three studies evaluated recovery time (in days) for SILC and CLC. The pooled mean difference was 0.01 (95% CI, -0.56 to 0.59), indicating no significant difference between the two groups (p = 0.73). Heterogeneity was low (chi² = 0.62, df = 2; I² = 0%). These findings suggest that recovery time is comparable between SILC and CLC. The results are shown in Figure 4.

Postoperative Pain

**Figure 5:**
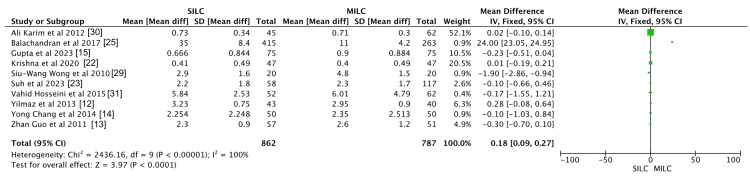
Meta-analysis comparing postoperative pain between single incision laparoscopic cholecystectomy and conventional laparoscopic cholecystectomy.

Ten studies evaluated postoperative pain scores in the SILC and CLC groups, including six randomized controlled trials (RCTs) and four non-randomized studies. Postoperative pain was marginally higher in the SILC group compared to the CLC group (mean difference, 0.18; 95% CI, 0.09-0.27; p < 0.001). However, significant heterogeneity was observed across studies (I² = 100%), indicating variability in the findings. Given the mix of study designs, variability in patient selection and treatment protocols may have contributed to the observed heterogeneity. A sensitivity analysis was conducted to assess the impact of study design on heterogeneity. These results are presented in Figure 5.

Wound Site Infection

**Figure 6:**
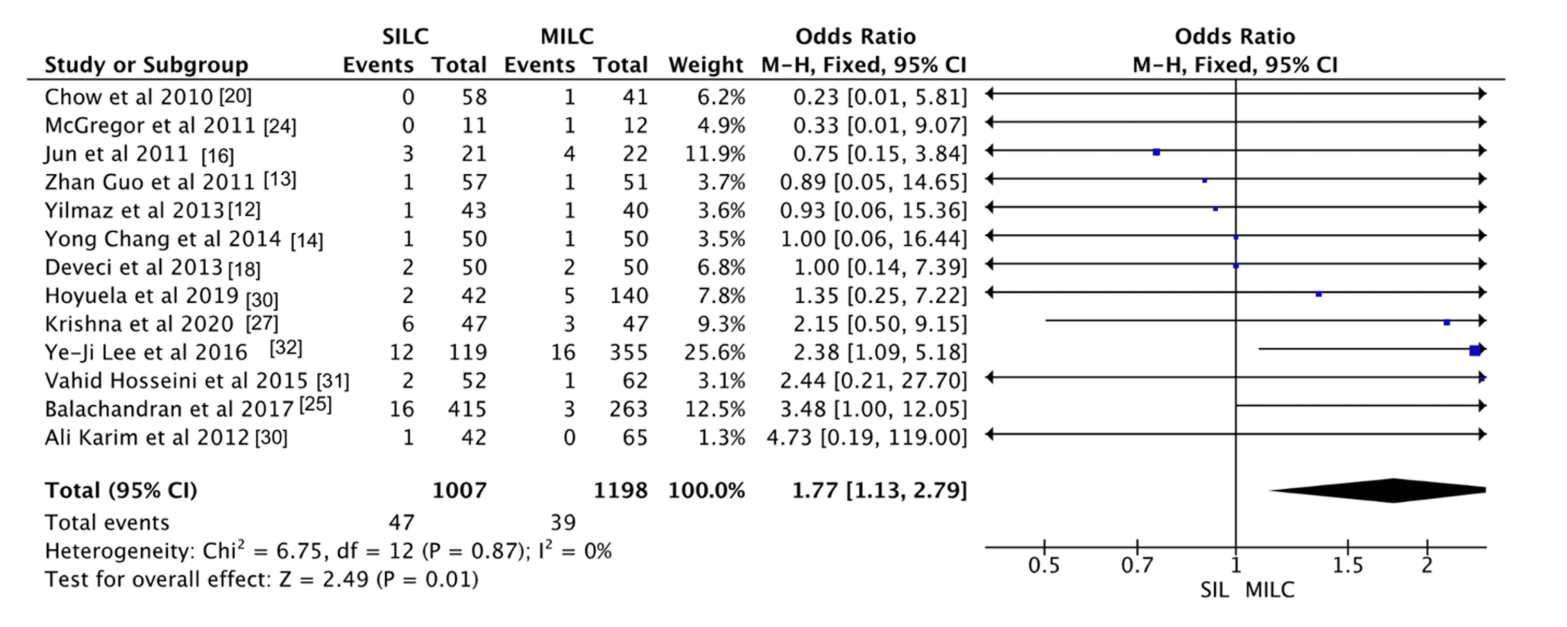
Meta-analysis comparing wound site infection between single incision laparoscopic cholecystectomy and conventional laparoscopic cholecystectomy.

Thirteen studies assessed wound infection rates in the SILC and CLC groups. The pooled odds ratio was 1.77 (95% CI, 1.30-2.79), indicating a significantly higher risk of wound infection in the SILC group compared to the CLC group. Heterogeneity was low (chi² = 0.67, df = 12; p = 0.87; I² = 0%). These findings are demonstrated in Figure 6.

Quality Assessment 

**Figure 7:**
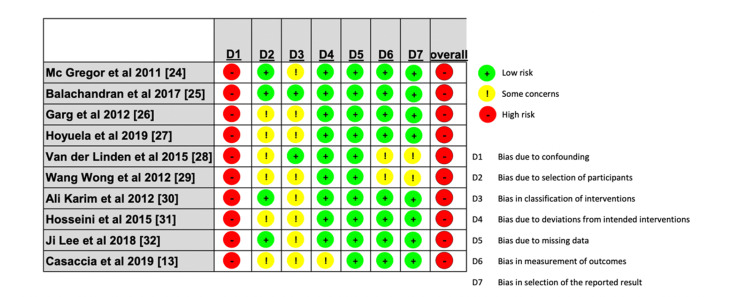
Detailed assessment of every study included in the systematic review using the ROBVIS tool. ROBVIS: risk of bias visualization tool [11]; D1: bias due to confounding; D2: bias due to selection of participants; D3: bias in classification of interventions; D4: bias due to deviations from intended interventions; D5: bias due to missing data; D6: bias in measurement of outcomes; D7: bias in selection of the reported result.

Data quality was assessed for non-randomized studies using ROBINS-1, and the results were visualized using ROBVIS, as shown in Figure 7.

**Figure 8:**
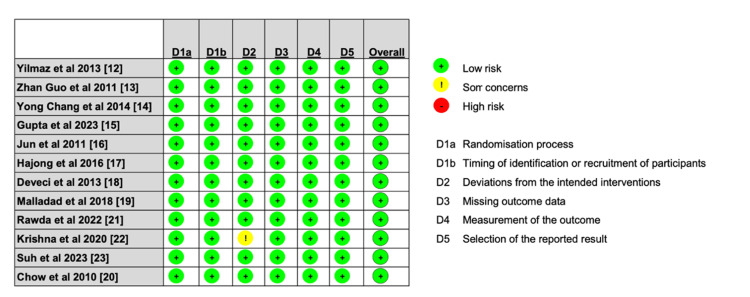
Detailed assessment of every study included in the systematic review using the ROBVIS tool. ROBVIS: risk of bias visualization tool [11] ; D1a: randomisation process; D1b: timing of identification or recruitment process; D2: Deviations from the intended interventions; D3: missing outcome data; D4: measurement of the outcome; D5: selection of the reported result

Data quality was assessed for randomized studies using ROB-2 as shown in Figure 8. 

Discussion

SILC is thought to provide improved cosmetic results, reduced operative trauma, fewer complications, quicker recovery, and reduced pain; however, its advantages over CLC are not definitively established. SILC may present challenges, including a steep learning curve for surgeons, technical complexity, the need for specialized equipment and training, and limited surgical field exposure, which could increase the risk of bile duct injury, similar to the initial experiences with CLC [16,32].

Hospital Stay

Our analysis showed a marginally shorter hospital stay in the CLC group, as evidenced by the pooled mean difference of 0.16 days (95% CI, 0.16-0.28). This difference could be attributed to the lower conversion rates observed in the CLC group, where fewer cases required escalation to open procedures compared to SILC. A previous study found no statistically significant difference in postoperative hospital stay between the two surgical techniques [33].

Postoperative Pain

This study demonstrated a significant difference in postoperative pain scores between SILC and CLC, with a mean difference of 0.18 (95% CI, 0.09-0.27), indicating slightly higher pain scores in the SILC group. This outcome is consistent with previous studies. For instance, Rosemurgy et al. noted greater pain complications in the single-incision group compared to a concurrent four-incision laparoscopic cholecystectomy group [34]. Similarly, Poon et al. found no benefit in pain reduction with fewer-port versus four-port laparoscopic cholecystectomy [35]. While incision length has been considered a factor in postoperative pain, other possible contributors, such as increased traction at the incision site or tissue manipulation, may also play a role. However, there is limited literature directly comparing these factors, and further research is needed to establish a definitive correlation.

Wound Site Infection

In the SILC group, 47 cases of infection were reported, while the CLC group had 39 cases of postoperative wound infections. An unpublished randomized trial by Navarra et al. suggests that the single, larger umbilical incision may have contributed to a higher rate of umbilical hernias in their patients. Other researchers have raised concerns that increased tissue trauma associated with a larger port site could elevate the risk of infection, delayed wound healing, and herniation [36,37]. In our opinion, the increased incidence of infections and complications in the SILC group may be partly attributed to the learning curve associated with the technique. SILC requires more advanced skills and precision compared to traditional multi-port laparoscopic surgery. Another possible explanation is that umbilical wounds may inherently be more vulnerable to infection. However, further research is needed to determine whether incision size plays a significant role in the observed differences in wound complications.

Limitations

Surgeon experience with SILC could also have contributed to outcome variability, particularly in the early stages. Additionally, our study exhibited high heterogeneity, indicating substantial variability in the data. This variability is a limitation and suggests that further investigation is needed in future studies to identify potential sources and improve the reliability of findings. Furthermore, the absence of standardized postoperative protocols and follow-up care limits the ability to draw definitive conclusions. Future studies should include longer follow-up periods and more detailed technical information to enable more comprehensive comparisons.

## Conclusions

This study provides valuable insights into the comparison between SILC and CLC. While SILC may offer improved cosmetic outcomes, it presents challenges such as higher postoperative pain scores, increased infection rates, a steeper learning curve for surgeons, longer hospital stays, and more complications. However, CLC demonstrated marginally shorter hospital stays and fewer complications in some aspects. Further studies with larger sample sizes, longer follow-up periods, and more standardized techniques are needed to fully determine the benefits and limitations of SILC.
